# ﻿Descriptions of two new stick insect species of *Cnipsomorpha* Hennemann, Conle, Zhang & Liu (Phasmatodea) from China based on integrative taxonomy

**DOI:** 10.3897/zookeys.1176.75490

**Published:** 2023-08-22

**Authors:** Fangling Xu, Yingjie Jiang, Maofa Yang

**Affiliations:** 1 College of Forestry, Guizhou University, Guiyang, Guizhou 550025, China Guizhou University Guiyang China; 2 Institute of Entomology, Guizhou University, Guiyang, Guizhou 550025, China Guizhou Light Industry Technical College Guiyang China; 3 Research Center for Biodiversity and Natural Conservation, Guizhou University, Guiyang, Guizhou 550025, China Guizhou University Guiyang China; 4 Department of Light Industry & Chemical Engineering, Guizhou Light Industry Technical College, Guiyang, Guizhou 550025, China Guizhou Light Industry Technical College Guiyang China

**Keywords:** *28S*, Biodiversity, *COI*, morphology, taxonomy, stick insects

## Abstract

Accurate taxonomical identification is an extremely important basis for stick insect research, including evolutionary biology but also applied biology such as pest control. In addition, genetic methods are a valuable identification auxiliary technology at present. Therefore, this paper used morphological and molecular data to investigate five stick insect specimens from the genus *Cnipsomorpha* in Yunnan, successfully identifying two new species: *Cnipsomorphayunnanensis* Xu, Jiang & Yang, **sp. nov.** and *C.yuxiensis* Xu, Jiang & Yang, **sp. nov.** A phylogenetic tree was constructed through their *28S* and *COI* genes in order to infer the phylogenetic position of the two new species. Photographs of the new species and a key to all known *Cnipsomorpha* species are provided.

## ﻿Introduction

*Cnipsomorpha* Hennemann, Conle, Zhang & Liu, 2008 (Phasmatodea: Clitumninae: Medaurini) represents a genus of stick insects that was published based on specimens from China, also including one undescribed species from Vietnam (Ho 2021). Fifteen species have been published to date: *C.apteris* (Liu & Cai, 1992), *C.bii* Ho, 2017, *C.colorantis* (Chen & He, 1996), *C.daliensis* Ho, 2017, *C.erinacea* Hennemann, Conle, Zhang & Liu, 2008, *C.inflexa* Ho, 2021, *C.jinpingensis* Ho, 2021, *C.kunmingensis* Chen & Pan, 2009, *C.nigromaculata* Ho, 2021, *C.nigrospina* Ho, 2021, *C.polyspina* Ho, 2021, *C.serratitibia* Ho, 2021, *C.trituberculata* Ho, 2021, and *C.viridis* Ho, 2021, all distributed in Yunnan (Liu and Cai 1992; Chen and He 1996; Hennemann et al. 2008; Chen and Pan 2009; Ho 2017, 2021) and *C.maoershanensis* Ho, 2017 found only in Guangxi (Ho 2017).

Reconstructing the phylogenetic relationships of phasmatodeans has been based mostly on molecular data in many previous studies (Sandoval et al. 1998; Wheeler et al. 2001; Whiting et al. 2003; Terry and Whiting 2005; Kômoto et al. 2010; Bradler et al. 2014; Simon et al. 2019) which facilitated the revisions that were mostly based on traditional morphological classification (Glaw et al. 2019; Madeira-Ott et al. 2020; Bank et al. 2021a; Cumming et al. 2021). *Cnipsomorpha* is considered to pertain to the clade Clitumninae sensu Hennemann et al. (2008), that was first corroborated by molecular results in Simon et al (2019) and subsequently recovered by Tihelka et al. (2020). Therefore, more research is needed.

In this study, we found two new species *Cnipsomorphayunnanensis* Xu, Jiang & Yang, sp. nov. and *C.yuxiensis* Xu, Jiang & Yang, sp. nov., and carried out the molecular study of *Cnipsomorpha* for the first time. We demonstrate that *Cnipsomorpha* together with *Parapachymorpha* and *Spinoparapachymorpha* form the sister group to Pharnaciini.

## ﻿Materials and methods

### ﻿Sample collection and treatment

A total of four specimens was collected in China in 2015 by net-sweeping of ferns, ﬁxed in 75% ethanol, and brought back to the laboratory for storage in a -80 °C refrigerator. Images were taken using a Canon EOS 60D suite (Canon Inc., Tokyo, Japan). Morphological terms follow Bragg (1997) and Ho (2021), and relevant literature information was obtained from the Phasmida Species File database (Brock et al. 2021). The type specimens were deposited at the College of Forestry, Guizhou University, China.

### ﻿DNA extraction, PCR, and sequencing

Genomic DNA was extracted from femoral tissue using the Ezup Column Animal Genomic DNA Purification Kit (Sangon Biological Engineering Co., LTD, Shanghai, China; hereafter, SG). First, reagents were prepared according to the instructions. 0.2 mg of muscle tissue was taken, placed in a 1.5 mL centrifuge tube, and 80 μl ACL buffer (all buffers from Ezup Column Animal Genomic DNA Purification Kit) were added, and the tube placed in a bath at 56 °C for 0.5 h. 100 μl ACL buffer and 20 μl Proteinase K were added and again placed in the water bath at 56 °C for 1 h; the subsequent operations were carried out according to the instructions of the kit.

The *28S* and *COI* target fragments were ampliﬁed and sequenced using the PCR primers listed in Table 1. Reagents were added for the PCR reaction test with reference to 2X SanTaq PCR Mix (SG): 15 μL 2X SanTaq PCR Mix, 1 μL forward primer, 1 μL reverse primer, 2 μL template, and 11 μL sterilized dd H_2_O. PCR employed the following temperature cycles through a KQ60 thermal cycler (Hangzhou Lattice Scientific Instrument Co., LTD, Zhejiang, China): 5 min of initial denaturation at 94 °C, followed by 35 cycles of 30 s of denaturation at 90 °C, 30 s of alignment at 45 °C, and 30 s of extension at 72 °C, finishing with 10 min of 72 °C and stored at 4 °C. High-quality PCR products (no clean-up) were bidirectional sequenced by Sanger sequencing technology of SG.

**Table 1.** Primers used for PCR and sequencing.

Gene	Primers*	Sequences(5’–3’)	References
*COI*	C1-J-2195	TTGATTTTTTGGTCATCCAGAAGT	Simon et al. 1994
*COI*	TL2-N-3014	TCCAATGCACTAATCTGCCATATTA	Simon et al. 1994
*28S*	*28S* Road 1a	CCCSCGTAAYTTAGGCATAT	Terry and Whiting 2005
*28S*	*28S* Road 4b	CCTTGGTCCGTGTTTCAAGAC	Terry and Whiting 2005

* Their annealing temperature is 45 °C.

### ﻿Phylogenetic reconstruction

The obtained sequences from SG were viewed, checked, and edited by BIOEDIT v. 7.0.9.0 (Alzohairy 2011), then compared and edited through DNAMAN v. 6.0.3.99 to obtain high-quality sequences (Wang 2016). A BLAST search was used to compare high-quality sequences with the NCBI database (Li et al. 2014). The sequences were uploaded to GenBank and the accession numbers are provided in Table 2. Then, all available sequences of Clitumninae were downloaded from the NCBI database (Table 2; Law and Crespi 2002; Whiting et al. 2003; Buckley et al. 2009; Djernæs et al. 2011; Bradler et al. 2014, 2015; Song et al. 2015; Robertson et al. 2018; Bank et al. 2021b).

**Table 2.** Sequence information and GenBank accession numbers. New species are in bold.

Subfamily	Tribe	Species	*28S*	*COI*
Clitumninae	Medaurini	***Cnipsomorphayunnanensis***	**MZ486038**	**MZ435970**
Clitumninae	Medaurini	***Cnipsomorphayuxiensis***	**MZ486045**	**MZ435977**
Clitumninae	Clitumnini	*Ramulusthaii*	FJ474166.1	FJ474322.1
Clitumninae	Clitumnini	*Ramulusartemis*	KJ024395.1	
Clitumninae	Clitumnini	*Ramulusnematodes*	MN925497.1	MN925741.1
Clitumninae	Clitumnini	*Cuniculinacuniculus*	MK291890.1	
Clitumninae	Clitumnini	*Lobofemorascheirei*	MN925432.1	
Clitumninae	Clitumnini	*Rhamphophasmaspinicorne*	MK291839.1	
Clitumninae	Medaurini	*Medauroideaextradentata*	KT426670.1	KT426637.1
Clitumninae	Medaurini	*Medauromorphafoedata*	MN925435.1	MN925689.1
Clitumninae	Medaurini	*Parapachymorphaspinigera*	MK291850.1	
Clitumninae	Medaurini	*Spinoparapachymorphaspinosa*	MK291851.1	
Clitumninae	Pharnaciini	*Pharnaciaponderosa*	MN925409.1	MN925665.1
Clitumninae	Pharnaciini	*Phobaeticusserratipes*	MK291836.1	
Clitumninae	Pharnaciini	*Phobaeticusfoliatus*	MN925378.1	MN925636.1
Clitumninae	Pharnaciini	*Phobaeticusheusii*	AY125324.1	
Clitumninae	Pharnaciini	*Phobaeticuskirbyi*		KT426649.1
Clitumninae	Pharnaciini	*Tirachoideawestwoodii*	MK291837.1	
Timematinae	Timematini	*Timemacalifornicum*	KM853347.1	AF410061.1

All sequences were aligned by MAFFT v. 7.149 (Katoh and Standley 2014), then trimmed with the GBLOCKS v. 0.91b (Talavera and Castresana 2007). The best-fit substitution model of reconstructed *28S* and *COI* trees was selected using the AIC criterion with JMODELTEST v. 2.1.7 (Darriba et al. 2012). According to the divergence between *Timema* and the Euphasmatodea that occurred more than 120 Myr ago (Simon et al. 2019), *Timema* was selected as outgroup. Based on the best-fit substitution model with 1000 bootstrap replicates using MEGA v. 7.0.26, maximum likelihood (ML) trees for each gene were reconstructed separately (Kumar et al. 2016) and visualized in FIGTREE v. 1.4.3 (https://github.com/rambaut/figtree).

## ﻿Results

### ﻿Taxonomic account

**Clitumninae Brunner von Wattenwyl, 1893**

**Medaurini Hennemann & Conle, 2008**

#### ﻿ Cnipsomorpha

Taxon classificationAnimaliaPhasmatodeaPhasmatidae

Hennemann, Conle, Zhang & Liu, 2008

B90647B8-B7D8-57BE-AF84-B1DC01C60462

##### Type species.

*Cnipsomorphaerinacea* Hennemann, Conle, Zhang & Liu, 2008.

##### Distribution.

Guangxi, Yunnan, China.

##### Note.

The two new species small to medium in size. Spinose. Apterous. The head is round or rectangular, with spines. Occiput raised, with spines. The antennae are short, shorter than the femoral segment of the forelegs, with distinct segments. Thoracic spines or tubercles. Pronotum is nearly trapezoidal. Meso- and metapleurae with small spines. Abdomen cylindrical, with triangular extension posterolaterally of abdominal terga II–VII in females, inconspicuous in males. In females, the sternum VII has an distinct praeopercular organ, the posterior edge of the anal segment is slightly concave, and the male’s 10^th^ abdominal segment is dorsally divided into two semi-tergites. This combination of characteristics distinguishes it from species of other genera and determines that the new taxa belong to *Cnipsomorpha*.

#### ﻿ Cnipsomorpha yunnanensis sp. nov.

Taxon classificationAnimaliaPhasmatodeaPhasmatidae

FF72105F-1EC0-5988-BCF3-E0494A810DE6

https://zoobank.org/DD51B013-D5F5-43CB-8760-14D47F5C4FCB

Fig. 1

##### Type material.

***Holotype*.** One Female. Fenshuiling Nature Reserve, Jinping County, China, alt. 2100 m, 18–19 May 2015, leg. Bin Yan. Specimen code: YNJP150517001. Specimen used for DNA extraction.

##### Differential diagnosis.

*Cnipsomorphayunnanensis* sp. nov. is similar to *Cnipsomorphaerinacea* Hennemann, Conle, Zhang & Liu, 2008 (Hennemann et al. 2008). In *C.yunnanensis* sp. nov., the vertex of the head has only two pairs of spines, where *C.erinacea* has more than two. In *C.yunnanensis* sp. nov., the mesonotum is not convex or swollen, and is armed with two pairs of spines and three pairs of tubercles, whereas in *C.erinacea* the mesonotum is convex, swollen, and armed with twelve prominent spines. Finally, in *C.yunnanensis* sp. nov., the middle parts of the terga II–IV are not raised and are armed with a single pair of spines in the posterior region while terga I–IX are expanded posterolaterally and are almost of the same length as the abdomen width; those of *C.erinacea* are raised and armed with several prominent spines, and shorter than the abdomen width.

*Cnipsomorphayunnanensis* sp. nov. also similar to *C.jinpingensis* Ho, 2021. In *C.yunnanensis* sp. nov., the lateral margins of the pronotum possess a spine medially, and the posterior margin of the anal segment exhibit a large trapezoidal concave edge. But in *C.jinpingensis*, the lateral margins of pronotum are without a spine medially, and the posterior margin of anal segment bear two to three small emarginations (Ho 2021).

##### Description.

**Figure 1.**
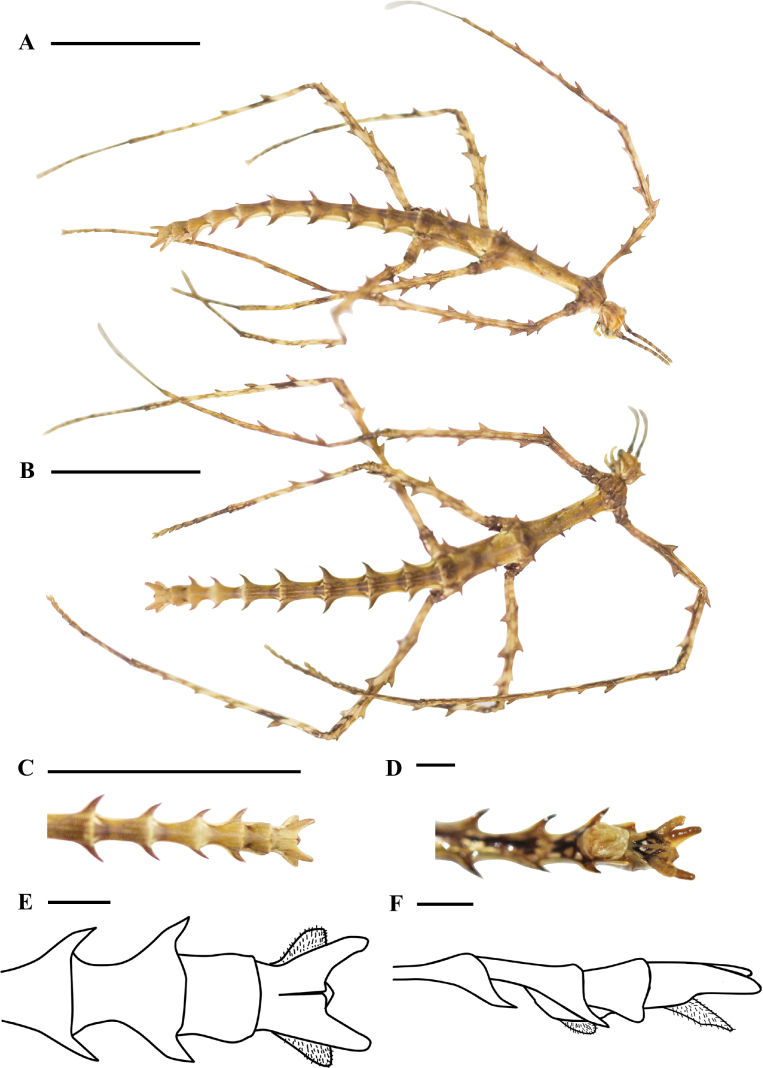
Features of *Cnipsomorphayunnanensis* sp. nov. Female **A** body, lateral view **B** body, dorsal view **C** hind part of abdomen, dorsal view **D** hind part of abdomen, ventral view **E** end of abdomen, dorsal view **F** end of abdomen, lateral view. Scale bars: 10 mm (**A–C**); 1 mm (**D–F**).

**Female** (Fig. 1). Apterous. Body slender, with some sparse, small granules. General color of specimen in alcohol is yellow. Expanded terga I–IX are curved and spine-like. Legs with serrations.

***Head*** oval, with irregular granules, without distinct tubercles. Compound eyes are spherical and protruding. There is one pair of spines on the vertex of the head, the apex leans forward. Occiput significantly reduced, with one pair of spines. Antennae filamentous, distinctly segmented, 18 segments, bristly, shorter than profemora, scapus oval, strongly compressed basally, longer than the third segment, third segment longer than the pedicellus, and apical segment roughly the same as the third segment.

***Thorax*** slender, midline distinct, with sparse granules and spines. Pronotum shorter than head, midline distinct, and slight bulge on both sides of the midline, one pair of spines in the posterior region, interspersed with one or two spine-like tubercles. Mesonotum longer than the head and pronotum combined, with one pair of small tubercles in the anterior region, two pairs of spines in the posterior region, one pair of tubercles near both sides in the middle region, and one pair of spine-like tubercles in the posterior region. Metanotum shorter than the mesonotum, anterior margins lacking spines, middle region with two pairs of thorns, posterior region with one pair of thorns tilted backward, and rear edge near both sides with one pair of small thorns. Meso- and metapleurae with granules, and each with one spine before the base segment of the mesocoxa. Meso- and metasternum with granules.

***Abdomen*** slender, with sparse granules, terga with spines, apex of these spines towards the apex of abdomen, terga II–IX with expanded posterolateral angles (Fig. 1C, D). Median segment shorter than metanotum, and width greater than length. Terga I–V with one pair of spines posteromedially. The 1^st^–5^th^ pairs of spines gradually increase. Fifth pair of spines smaller than the 2^nd^ pair of spines but larger than the 1^st^ pair of spines. Terga II–VIII with expanded posterolateral angles, gradually increasing in size from the 2^nd^–4^th^ pair, the 5^th^–7^th^ gradually decreasing in size, 8^th^ pair larger than the 7^th^ pair but smaller than the 6^th^ pair, 9^th^ pair distinctly expanded, the shape of spine-like tubercles. Sternum VII with an indistinct preopercular organ (Fig. 1D, F). Anal segment longer than tergum IX and shorter than tergum VIII, posterior margins with a large trapezoidal concave edge, apex rounded. Cerci flat, leaf-shape, apex rounded (Fig. 1E). Subgenital plate with one spine-like tubercle, extending approximately to the posterior margin of tergum IX (Fig. 1E).

***Legs*** slender and with serrations. Profemora incurved basally, with two serrations of the anterdorsal carina, without serrations of the posterodorsal carina, with three serrations of the antero- and posteroventral carina. Antero- and posterodorsal carina with three serrations of the mesofemora, middle serration the largest, antero- and posteroventral carina with three serrations roughly the same size. Antero- and posterodorsal carina with three serrations of the metafemora, middle serration the largest, antero- and posteroventral carina with three serrations, middle serration the largest. All tibial anterodorsal and ventral carina unarmed. Posterodorsal carina of protibiae with six serrations, gradually smaller towards the apex, interspersed with large spines occasionally. Posterodorsal carina of meso- and metatibiae with two serrations. Mesobasitarsus shorter than the rest combined, pro- and metabasitarsus longer than the rest combined.

**Male** and **eggs** unknown.

**Measurements** are provided in Table 3.

**Table 3.** Measurements (mm) of *Cnipsomorphayunnanensis* sp. nov. and *C.yuxiensis* sp. nov.

	*C.yunnanensis*, Holotype, female	*C.yuxiensis*, Holotype, female	*C.yuxiensis*, Allotype, male
Body	33.00	47.00	31.50
Head	1.90	3.83	2.98
Antennae	3.18	5.39	8.09
Pronotum	1.38	3.12	2.20
Mesonotum	5.81	7.09	6.24
Metanotum	4.56	3.90	4.68
Median segment	1.11	2.28	2.49
Profemur	13.13	12.76	17.03
Mesofemur	8.39	7.73	11.80
Metafemur	10.92	9.01	11.97
Protibiae	16.18	13.10	18.09
Mesotibiae	8.85	9.86	12.59
Metatibiae	13.41	13.05	12.20

##### Distribution.

Jinping, Yunnan, China.

##### Etymology.

This species is named after the province type locality, Yunnan, China.

##### Comments.

Only known from one female; the specimen is now missing the mesofemur due to DNA extraction. This new species is flatter than other species in this genus, which may have been caused by insufficient nutrition prior to being collected.

#### ﻿ Cnipsomorpha yuxiensis sp. nov.

Taxon classificationAnimaliaPhasmatodeaPhasmatidae

14DF57C2-1738-518B-BB21-4BD479F96739

https://zoobank.org/C6CA7BAF-4697-42AA-A3B7-D6B98E97F281

Fig. 2

##### Type material.

***Holotype*.** One female. Ailaoshan Primeval Forest, Gasha, Xinping, Yuxi, Yunnan, China, alt. 2400 m. 8 May 2015. Collectors: Bin Yan, Yunfei Wu. Specimen code: YNYX150508001-1. ***Allotype*.** One male. Same data as holotype. Specimen code: YNYX150508001-2. Specimen used for DNA extraction. ***Paratype*.** One female. Jinshanyakou, Ailaoshan, Xinping, Yuxi, Yunnan, China. alt. 2377–2413 m, 17 May 2015, leg. Bin Yan. Specimen code: YNYX150507002.

##### Differential diagnosis.

The female of *C.yuxiensis* sp. nov. is similar to *C.trituberculata* Ho, 2021. In *C.yuxiensis* sp. nov., the anterodorsal and posterodorsal carinae of femora bear inconspicuous serrations, where *C.trituberculata* exhibits distinct serrations. In *C.yuxiensis* sp. nov., the seventh abdominal sternum bears an indistinct preopercular organ, where *C.trituberculata* has a distinct preopercular organ. In *C.yuxiensis* sp. nov., the female middle area of the mesonotum shows nine spine-like tubercles, where *C.trituberculata* has twelve. The male of *C.yuxiensis* sp. nov. is similar to the *C.viridis* Ho, 2021. In *C.yuxiensis* sp. nov., the sixth abdominal tergum is unarmed, where *C.viridis* has paired posterior medial spines. In *C.yuxiensis* sp. nov., the spines on the metanotum are not paired and are sparse, where *C.viridis* has0 paired posterior medial and pre-median spines.

##### Description.

**Figure 2.**
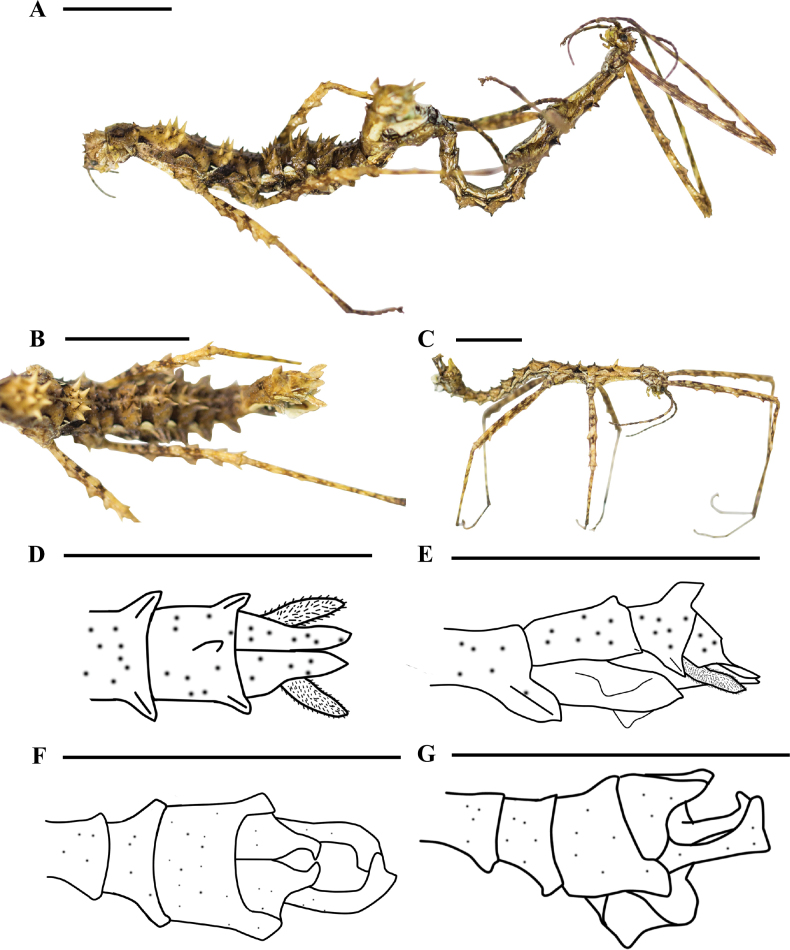
Features of *Cnipsomorphayuxiensis* sp. nov. Female (**A, B, F, G**) and male (**A, C–E**) **A** male and female, in copulation, lateral view **B** body, dorsal view **C** body, dorsal view **D** end of abdomen, drawing of dorsal view **E** end of abdomen, lateral view **F** end of abdomen, dorsal view **G** end of abdomen, lateral view. Scale bars: 10 mm.

**Female** (Fig. 2A, B, D, E). Slender and granulated. Apterous. The color of the specimen in alcohol is generally yellowish to brown, with black markings.

***Head*** nearly spherical, with sparse irregular granules. Compound eyes large, spherical, and protruding. Vertex of head with one pair of spines, apex pointed. Occiput without swelling, with six spine-like tubercles, one pair close to the edge, middle pair larger. Antennae filamentous, distinctly segmented, bristly, shorter than profemora. Scapus oval, longer than the third segment, third segment longer than the pedicellus.

***Thorax*** slender, with distinct granules. Pronotum shorter than head, slightly longer than wide, transverse sulcus in middle area, longitudinal sulcus indistinct, posterior margins raised, with a pair of spine-like tubercles, anterior and posterior regions slightly convex. Mesonotum longer than length of head and pronotum combined, mesonotum raised, anteromedially with eleven tubercles. Midline of mesonotum with seven spine-like tubercles, six of which form three pairs, one tubercle in the area between two pairs of large spine-like tubercles, the first smaller and the remaining four on both sides. Posterior region of mesonotum with one pair of spine-like tubercles. Middle area of metanotum raised, with three pairs of spine-like tubercles, two pairs near the midline, one pair in the middle area near the margins, rear pair largest, and rear margins with one pair of spine-like tubercles. Meso- and metapleurae and meso- and metasternum with granules.

***Abdomen*** slender with distinct granules, and with spine-like tubercles whose apexes lean towards the apex of abdomen. Median segment shorter than metanotum, with two pairs of very small spine-like tubercles, with distinct granules.

Terga II–V with three pairs of spine-like tubercles, gradually increasing in size to tergum IV (largest), middle pair closest to midline, anterior pair next closest to midline, posterior pair closest to posterior margins (Fig. 2B). Tergum V tubercles smaller than those of tergum II. Posterior margins of tergum VIII with one spine-like tubercle, and posterior area of tergum IX with parallelogram-like bulge (Fig. 2E). Expanded posterolateral angles of terga I–IX spade-like, 1^st^ indistinct, 2^nd^–7^th^ gradually increasing in size, 8^th^–9^th^ gradually decreasing in size. Sternum VII with indistinct preopercular organ. Anal segment longer than tergum IX but shorter than tergum VIII, posterior margins with deep concavity, and posterior area dilated with two long, distinct, spade-like segments. Subgenital plate boat-like, with three carina, each ridge (except middle one) with a peak, apex pointed but does not surpass posterior margins of anal segment. Cerci distinct, slightly surpassing posterior margins of anal segment, apex blunt (Fig. 2D, E).

***Legs*** lender and with serrations, all antero- and posterodorsal carina of femora apex extend and pointed. All coxa with tubercles, one or two small spine-like tubercles (or none). Profemora incurved basally, antero- and posterodorsal carina with five indistinct peaks, antero- and posteroventral carina with five serrations, base spine very small, with rest basically the same size. Antero- and posterodorsal carina of mesoforma with three peaks, antero- and posteroventral carina with three serrations. Antero- and posterodorsal carina of metaforma with five peaks, antero- and posteroventral carina with three serrations. All tibiae unarmed, with two or three peaks, except for prominent mesotibial bulge, with rest relatively shallow. Each basitarsus shorter than the rest of the tarsus combined.

**Male** (Fig. 2A, C, F, G) more slender than female, granulated. Apterous. Color of specimen in alcohol is generally yellowish to brown, with black markings.

***Head*** nearly spherical, with sparse irregular granules. Vertex of head with one pair of spines. Compound eyes spherical and protruding. Occiput without swelling, with four pairs of spine-like tubercles, two close to the middle, one each on anterior and posterior margins, one pair (different sizes) in posterior area. Antennae filamentous, distinctly segmented, bristly, shorter than profemora; scapus shorter than third segment but longer than pedicellus.

***Thorax*** slender with irregular granules. Pronotum shorter than head, anterior margins with distinct transverse sulcus, middle area raised, posterior area slightly sunken, and posterior area raised with one pair spine-like tubercles. Mesonotum longer than head and pronotum combined. Middle and posterior areas with one pair each of spine-like tubercles, one pair largest in the middle. Metanotum shorter than mesonotum, and tubercles same size as mesonotum. Meso- and metapleurae with granules. Meso- and metasternum with granules and sparse, irregular, spine-like tubercles.

***Abdomen*** slender, cylindrical, with granules, and with irregular black ring. Terga II–V with one pair of spine-like tubercles posteromedially, their apexes directed towards the apex of abdomen (Fig. 2C). Terga I–IX with expanded posterolateral angles with spine-like shape and pointed apex. Posterior margins of tergum IX expand outward and backward. With Y-shaped vomer of abdominal segment IX, and apex exceeding the posterior margins of anal segment (X) (Fig. 2F, G). Anal segment with deep concavity, and posterior area dilated as two distinct segments, the two side plates bent inwards, their apexes in contact. Poculum with pocket-like shape, middle area with peak, and apex blunt and not exceeding the posterior margins of anal segment (Fig. 2G).

***Legs*.** slender, with granules. All coxae of legs with one or two small spine-like tubercles (or none). Profemora incurved basally, dorsal carina wavy but indistinct, antero- and posteroventral carina with four small serrations, posteroventral carina serrations larger than anteroventral carina. Dorsal carina of meso- and metafemora wavy and distinct, antero- and posteroventral carina with three serrations, larger serrations in posteroventral carina. Tibiae without distinct serrations, wavy but indistinct. Each basitarsus shorter than the rest of them combined.

**Measurements** are given in Table 3.

**Eggs** unknown.

##### Distribution.

Yuxi, Yunnan, China.

##### Etymology.

This species is named after the type locality, Yuxi, Yunnan, China.

##### Comments.

One of the females lacks the mesofemur due to DNA extraction. It is very rare to be able to collect the specimens in a mating state in the wild but fortunately we were able to do so in this case (Fig. 2A). The Y- shaped vomer of the male in this new species is quite different from that of all other species of *Cnipsomorpha*.

### ﻿Key to all known species of *Cnipsomorpha* (revised from Ho 2021)

Females

**Table:** 

1	Anterodorsal and posterodorsal carinae of femora with distinct serrations	**2**
–	Anterodorsal and posterodorsal carinae of femora unarmed or with indistinct serrations	**9**
2	Tibiae without serrations	***C.apteris***
–	Tibiae with serrations	**3**
3	Lateral margins of pronotum without spine medially	**4**
–	Lateral margins of pronotum with a spine medially	**6**
4	Posterior margin of anal segment with a deep emargination	***C.inflexa***
–	Posterior margin of anal segment with 2 or 3 small emarginations	**5**
5	Anteroventral and posteroventral carinae of tibiae with indistinct serrations	***C.jinpingensis***
–	Anteroventral and posteroventral carinae of tibiae without serrations	***C.colorantis***
6	Body length > 60 mm	***C.wenxuani***
–	Body length < 60 mm	**7**
7	Middle area of mesonotum with 6 pairs of medial spines	***C.trituberculata***
–	Middle area of mesonotum with 2 or 4 pairs of medial spines	**8**
8	Middle area of mesonotum with four pairs of medial spines	***C.serratitibia***
–	Middle area of mesonotum with 2 pairs of medial spines	***C.yunnanensis* sp. nov.**
9	Seventh abdominal sternum with indistinct preopercular organ	***C.yuxiensis* sp. nov.**
–	Seventh abdominal sternum with distinct preopercular organ	**10**
10	Preopercular organ is flattened	**11**
–	Preopercular organ is cylindrical	**13**
11	Preopercular organ posterior margin rounded	***C.kunmingensis***
–	Preopercular organ posterior margin emarginated	**12**
12	Preopercular organ posterolateral apices is blunt	***C.viridis***
–	Preopercular organ posterolateral apices is pointed	***C.polyspina***
13	Preopercular organ hump-like	***C.daliensis***
–	Preopercular organ cylindrical	**14**
14	Preopercular organ short, apically rounded and tubercle-like	***C.erinacea***
–	Preopercular organ elongate, apically pointed and knife-like	**15**
15	Pronotum without paired anterior medial spines	***C.nigromaculata***
–	Pronotum with paired anterior medial spines	***C.bii***

Males

**Table:** 

1	Cerci longer than anal segment	***C.colorantis***
–	Cerci shorter than anal segment	**2**
2	Body length > 60 mm	***C.wenxuani***
–	Body length < 60 mm	**3**
3	Median segment without posterior spines	**4**
–	Median segment with posterior spines	**8**
4	Pronotum without posterior medial spines	***C.daliensis***
–	Pronotum with posterior medial spines	**5**
5	Sixth abdominal tergites with paired posterior medial spines	***C.viridis***
–	Sixth abdominal tergites without paired posterior medial spines	**6**
6	Semi anal tergites strongly incurved	***C.inflexa***
–	Semi anal tergites weakly incurved	**7**
7	Abdomen without vomer	***C.nigromaculata***
–	Abdomen with vomer	***C.yuxiensis* sp. nov.**
8	Tibiae without serrations	***C.kunmingensis***
–	Tibiae armed with serrations	**9**
9	Metanotum without median spines	***C.jinpingensis***
–	Metanotum with paired median spines	***C.maoershanensis***

### ﻿Phylogenetic analysis

**Figure 3.**
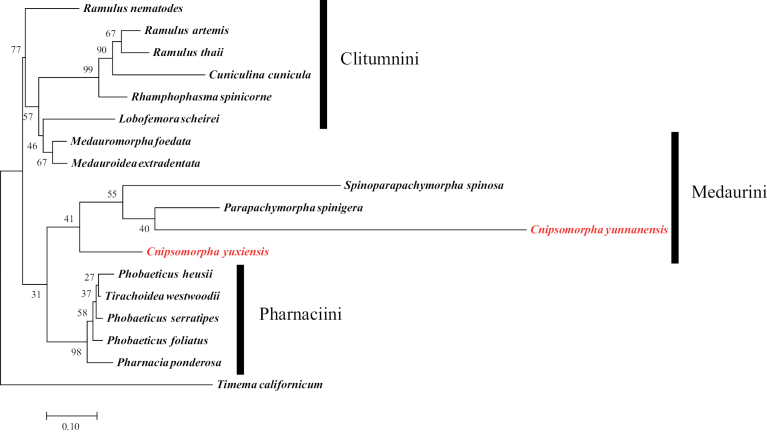
Maximum likelihood tree based on *28S* rDNA of available species of Clitumninae. GTR+G model with 1000 bootstraps. The outgroup is *Timemacalifornicum*.

**Figure 4.**
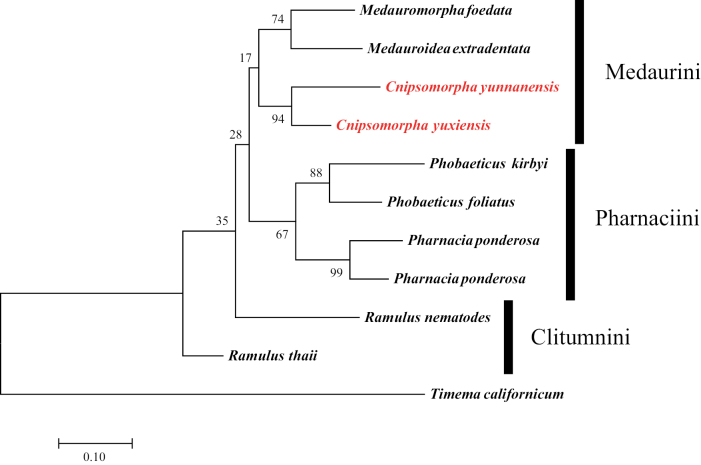
Maximum likelihood tree based on *COI* sequence data of available species of Clitumninae. The outgroup is *Timemacalifornicum*, GTR+I+G model with 1000 bootstraps.

**Figure 5.**
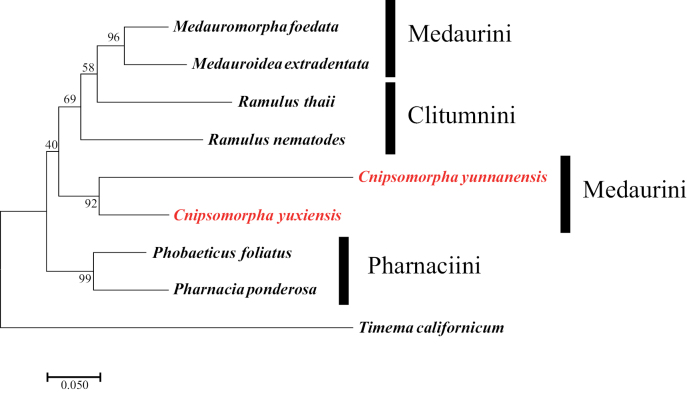
Maximum likelihood tree based on *28S* rDNA + *COI* of available species of Clitumninae. The outgroup is *Timemacalifornicum*, GTR+G model with 1000 bootstraps.

The best-fit substitution models were used to reconstruct the ML trees of *28S*, *COI*, and *28S* + *COI* are GTR+G (AIC value: 7301), GTR+G+I (AIC value: 6903) and GTR+G (AIC value: 11127). The reliability of the ML trees was tested by running 1,000 ultrafast bootstrap pseudoreplicates (Figs 3–5).

*Cnipsomorpha* form a clade with *Parapachymorpha* and *Spinoparapachymorpha* which together are the sister group to Pharnaciini (support values = 41) to several species of Clitumnini and Medaurini in the *28S*ML tree (Fig. 3), which confirms the work of Hennemann et al. (2008). Albeit, the *Cnipsomorpha* species have their own derived characteristics (autapomorphies) that define the taxon, namely the posterolateral extensions of the abdominal terga II–VII in females. In *Parapachymorpha* only the posterior expansion of the tergum VII of *P.jinpingensis* and the posterior expansion of the tergum VIII of *P.xishuangbannaensis* are present. The morphological characteristics of the two new species are quite different from those of *Parapachymorpha*.

In Fig. 4, *Cnipsomorpha* together with *Medauroidea* and *Medauromorpha* are the sister group of Pharnaciini. However, in *Medauroidea* and *Medauromorpha* there are no obvious spines or tubercles on the body surface of the latter, and without the posterolateral extensions of abdominal terga II–VII in females (Hennemann et al. 2008).

We think that the phylogenetic position of *Cnipsomorpha* should be closer to that of *Parapachymorpha* and *Spinoparapachymorpha*, because their body shape is more similar and the body shorter than that of *Medauroidea* and *Medauromorpha*. Moreover, in Fig. 5, the phylogenetic position of *Cnipsomorpha* is closer to the Clitumnini, and the *Parapachymorpha* and *Spinoparapachymorpha* belong to Clitumnini.

Besides, Medaurini can be divided into two parts as in the study of Bank and Bradler (2022). Medaurini I is close to Gratidiini, Medaurini II is close to Clitumnini, and the two new species of *Cnipsomorpha* belong to Medaurini I in the *28S*ML tree, which shows the same topology as in Bank and Bradler (2022). That is to say, *Cnipsomorpha* together with *Parapachymorpha* and *Spinoparapachymorpha* form the sister group to Pharnaciini, but *Cnipsomorpha* does not cluster together with the bulk of Medaurini species. Comprehensive research based on more taxa and data is necessary to corroborate these phylogenetic assumptions.

## ﻿Conclusions

We report two new species of *Cnipsomorpha*, *C.yunnanensis* sp. nov., and *C.yuxiensis* sp. nov., based on morphological characteristics, and generated molecular data for these two species. According to the results of our phylogenetic analysis, we can conclude that the phylogenetic position of the two new species is closer to *Parapachymorpha* than to other Medaurini, and that this clade is the sister group of Pharnaciini.

## Supplementary Material

XML Treatment for Cnipsomorpha

XML Treatment for Cnipsomorpha yunnanensis

XML Treatment for Cnipsomorpha yuxiensis

## ﻿Additional information

### Author ORCIDs

Fangling Xu https://orcid.org/0000-0002-4575-9155

Yingjie Jiang https://orcid.org/0000-0001-9083-2887

Maofa Yang https://orcid.org/0000-0002-5523-6825

### Data availability

All of the data that support the findings of this study are available in the main text.
